# Management of an Amyand’s hernia complicated by necrotizing soft tissue infection: a case report

**DOI:** 10.1093/jscr/rjag576

**Published:** 2026-08-14

**Authors:** Erin Major, Sadhvika Ramji, Jeffrey Emrich, Elizabeth Renza Stingone

**Affiliations:** Department of Graduate Medical Education, Rowan-Virtua School of Osteopathic Medicine, 1 Medical Center Drive, Stratford, NJ 08084, United States; Department of General Surgery, Virtua Hospital, 1660 Haddon Avenue, Camden, NJ 08103, United States; Department of General Surgery, Virtua Hospital, 1660 Haddon Avenue, Camden, NJ 08103, United States; Department of General Surgery, Virtua Hospital, 1660 Haddon Avenue, Camden, NJ 08103, United States

**Keywords:** inguinal hernia, scrotal abscess, biologic mesh, perforated appendicitis

## Abstract

Inguinoscrotal hernias account for ~2.7% of all inguinal hernias. Rare presentation limits the development of management guidelines for complex presentations of the condition. This case report reviews the management of an inguinoscrotal hernia containing perforated appendicitis with resultant necrotizing fasciitis. A 59-year-old male presented to the emergency department with worsening pain and edema in the right inguinal canal. Imaging revealed perforated appendicitis contained within an inguinoscrotal hernia, complicated by air in the abdominal wall consistent with necrotizing fasciitis. The patient was taken to the operating room for extensive wound debridement, appendectomy and orchiectomy with interval definitive hernia repair 48-h after index operation. This case demonstrates our success with a single 48-h operative interval, extended antibiotic course and biological mesh placement in the context of complicated inguinal hernia repair. Our experience with this patient should aim to inform future management of patients with similar conditions.

## Introduction

Accounting for less than 1% of inguinal hernias, containment of the vermiform appendix within an inguinal hernia sac, termed an Amyand’s hernia, is exceedingly rare [1–3]. Moreover, presentation of an Amyand hernia can mimic that of an incarcerated inguinal hernia making appropriate diagnosis challenging. As a result, many patients require direct intraoperative groin exploration for diagnostic confirmation. Intraoperative identification of small bowel contained within the sac can then further be complicated by incidental findings of appendicitis or bowel perforation. While complications such as these occur, they are exceptionally rare with appendicitis within the sac being encountered in only 0.11% of all cases [4, 5]. As such, management guidelines for perforated viscus contained within an inguinal hernia is nearly non-existent. To address this paucity of data, we aim to discuss our experience with anti-microbial therapy, use of biological mesh, and optimal time interval for definitive hernia repair in complicated inguinoscrotal hernias.

## Case report

**Figure 1:**
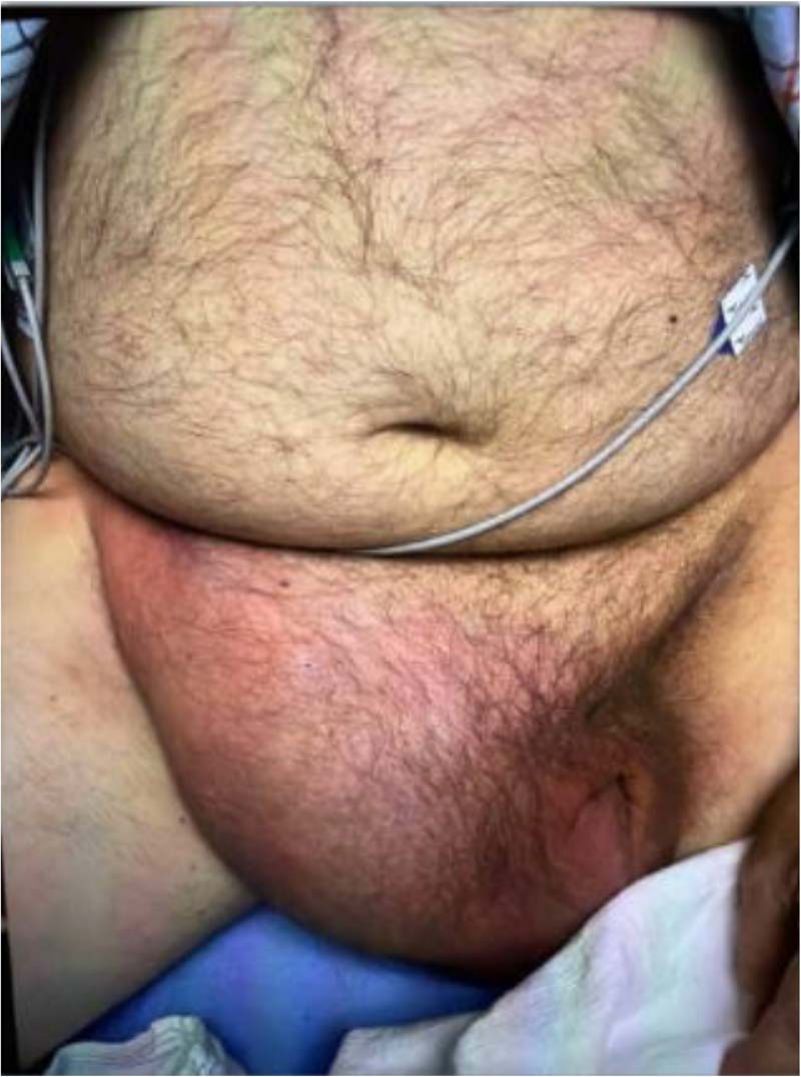
Physical exam findings.

A 59-year-old male with no significant past medical history presented with 1 week of subjective fever, decreased appetite, and increased pain and swelling in his right groin. He reported poor primary care follow-up but described a 10-year history of a small, reducible groin hernia, for which he has never seeked care. He denied any dysuria, hematuria, flank pain, nausea or vomiting. On evaluation, the patient was tachycardic with a non-reducible right inguinoscrotal hernia with edematous skin changes and crepitus (Fig. 1). The patient was kept nil per os (NPO), given maintenance fluids and sent for computed tomagraphy (CT) of the abdomen and pelvis without contrast.

**Figure 2:**
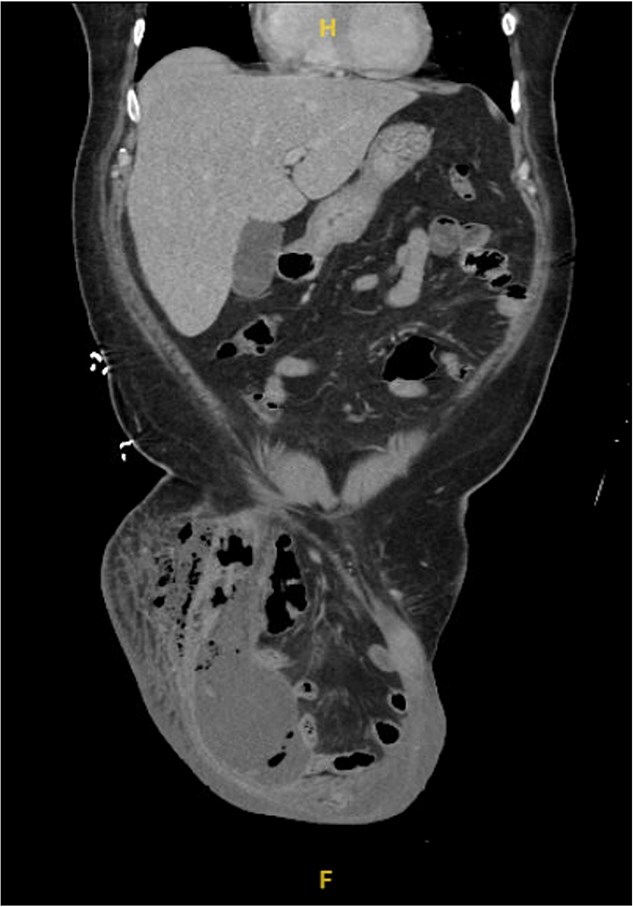
Coronal view of right inguinal hernia.

**Figure 3:**
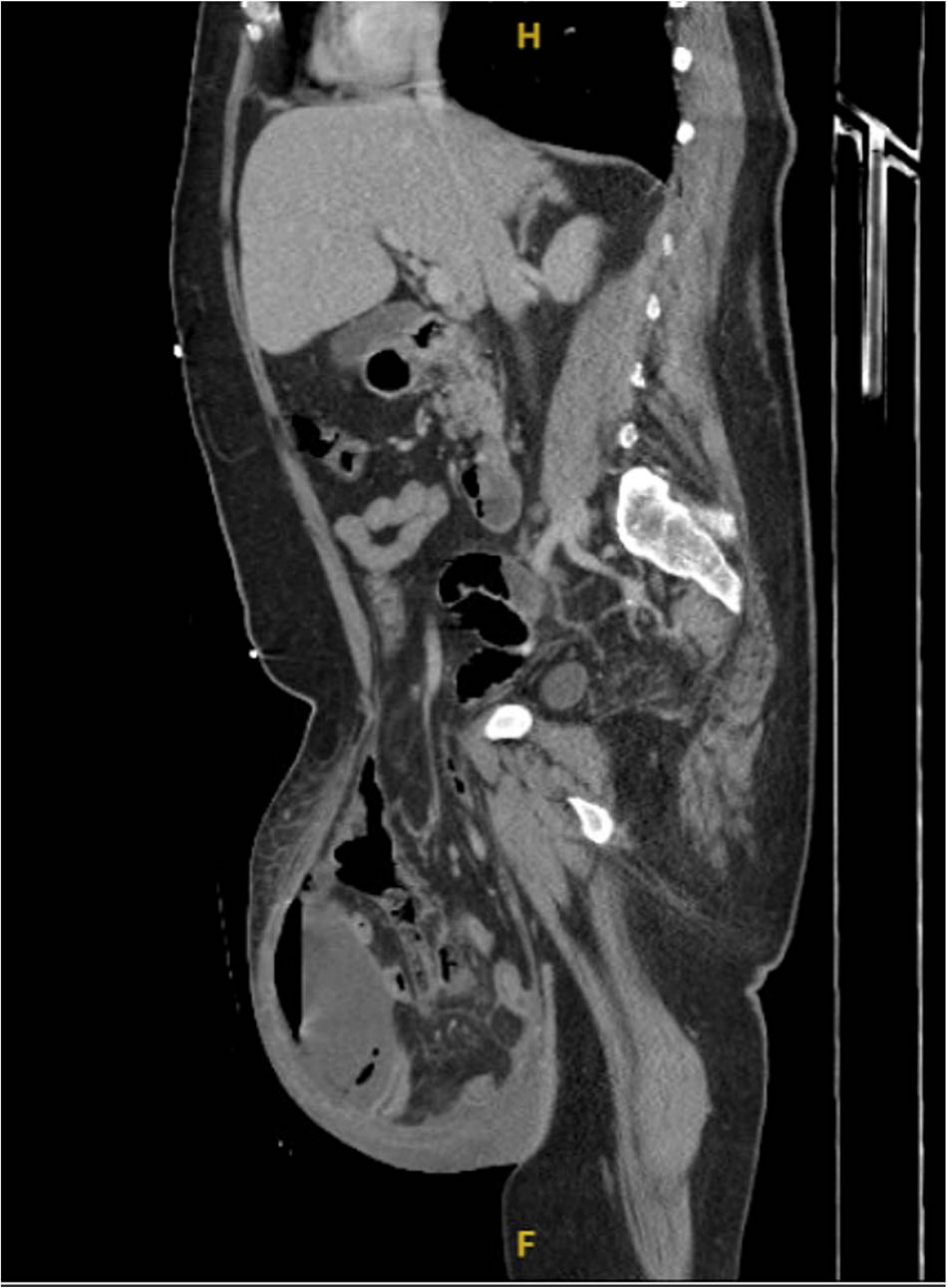
Sagittal view of the right inguinal hernia.

Imaging demonstrated a large right inguinoscrotal hernia containing portions of the distal ileum, terminal ileum, and proximal cecum measuring 17.3 × 13.5 × 22.4 cm (Fig. 2). Within this hernia, there was an 8.9 × 5.9 × 14.9 cm collection of gas, fluid, and air bubbles suggestive of an abscess complicated by necrotizing infection containing possible perforated appendicitis (Fig. 3). Labs correlated with imaging findings with severe leukocytosis (white blood cell count 34.78) and elevated lactate (2.5). Given these findings, the patient was started on broad spectrum IV Vancomycin and Zosyn while general surgery and urology were consulted for concern of incarcerated inguinoscrotal hernia.

**Figure 4:**
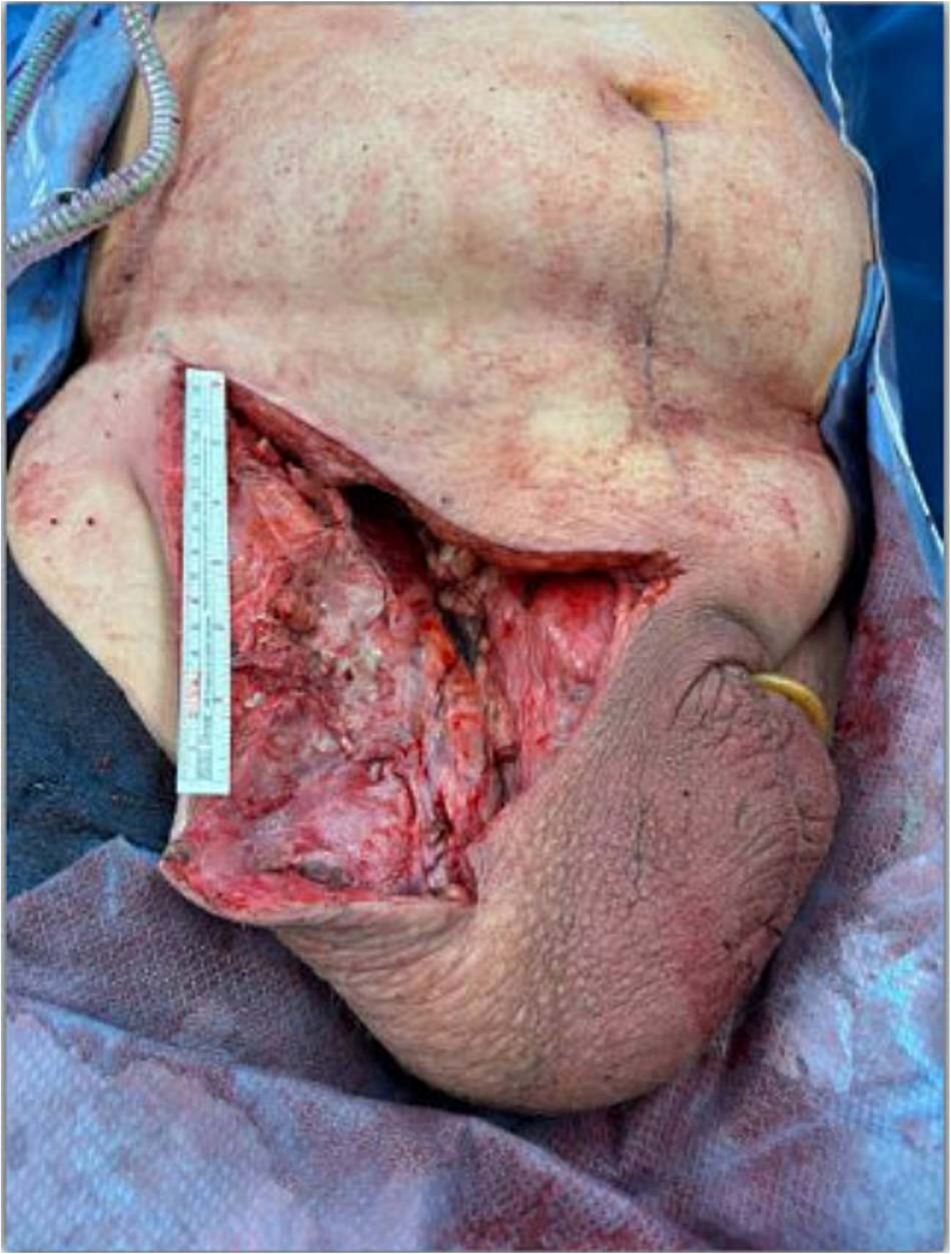
Surgical site post initial debridement.

The patient was consented and taken to the operating room for a right groin exploration. Following initial incision, ~200 cc of foul smelling pus was suctioned from the area with underlying extensive necrotic tissue involving skin, subcutaneous tissue, fascia and scrotal contents consistent with necrotizing fasciitis. All nonviable tissue was debrided sharply with the debrided area measuring 30 × 25 cm (Fig. 4). The appendiceal stump was then found to be severely inflamed and perforated, yet still maintained within the hernia sac, resulting in dense adhesions to the right testis ultimately requiring right orchiectomy. A subsequent appendectomy, herniated bowel reduction and primary scrotal abscess debridement were also performed with return of well perfused bowel to the abdominal cavity and closure of the inguinal ring. Once clean wound margins and viable bowel were confirmed, a negative pressure dressing was placed with planned return for definitive hernia repair at a later date.

Forty-eight-hours post initial debridement, the patient returned to the operating room for definitive inguinal hernia repair. A 10 × 5cm Transorb Medtronic mesh was placed for a Lichtenstein repair of the 7 cm inguinal canal defect. The patient had uncomplicated post-operative recovery without signs of post-operative infection or wound dehiscence. Following repair, wound cultures isolated *Enterobacter. Coli* and *Clostridium ramosum*, for which vancomycin was discontinued. The patient was simultaneously switched to an oral 2-week course of Augmentin 875–125 mg BID and discharged home 6 days after initial presentation. During his follow-up visit, 14 days post discharge, the patient was doing well with no complaints and without any recurrence of hernia on physical exam.

## Discussion

Subsequent interval debridement is not uncommon in Necrotizing Soft Tissue Infection (NSTI) management with current guidelines suggesting return to OR every 12–24 h, as needed for source control [6]. Recent data suggests shorter intraoperative intervals are associated with lower mortality rates, with 48-h intervals associated with increased mortality [6]. This patient, however, demonstrated adequate response to 48-h operative interval despite perforated, yet contained, viscus. While future investigations should further substantiate this, our experience with a single 48-h return for debridement suggests the possibility that in the presence of adequate antibiotic response, operative intervals need not be restricted to 12 h.

Decreased recurrence of inguinal hernias has driven the frequent use of tension-free mesh placement intraoperatively, yet choice of mesh remains up to defect characteristics and surgeon preference [7]. Despite biologic mesh recently being found to have higher recurrence rates in a contaminated field, this patient responded adequately to the Transorb Medtronic biologic mesh [8]. The self-gripping quality of this mesh may have ameliorated biologic associated inferior recurrence rates and provided satisfactory repair in this 7 cm inguinoscrotal hernia defect [9]. Overall, the biologic mesh provided sufficient inguinoscrotal hernia repair and should be considered in future patients.

Necrotizing soft tissue infections (NSTI) secondary to perforated appendicitis within an inguinal hernia sac provide unique challenges to surgical management with inherent gastrointestinal and genitourinary flora contamination making decolonization challenging. Generally, broad spectrum beta-lactams in conjunction with vancomycin and linezolid have been proposed as appropriate therapies for NSTI, yet duration of antimicrobial therapy is not as clearly defined [6, 10]. This patient received antibiotic therapy for a total of 21 days with successful resolution of infection. Successful individual response to this antibiotic course in this patient suggests that complicated inguinoscrotal hernias may be responsive to a 3-week course of initial broad spectrum followed by targeted beta-lactamase antibiotic therapy.

Our experience with this patient suggests that beta-lactamases may provide appropriate microbial coverage in conjunction with 48-h operative interval and biologic mesh placement. Through discussion of this patient’s excellent surgical care and overall recovery, we hope to better inform future management of complicated Amyand hernias.
